# Supplementary material for: Impact of Homologous Recombination on Core Genome Evolution and Host Adaptation of Pectobacterium parmentieri
Source: Genome Biol Evol. 2024 Feb 22;16(3):evae032. doi: 10.1093/gbe/evae032 (PMC10946231; doi:10.1093/gbe/evae032)
Supplement: evae032_Supplementary_Data [file evae032_supplementary_data.zip › SupplementaryFigures_S1_S2.pdf]

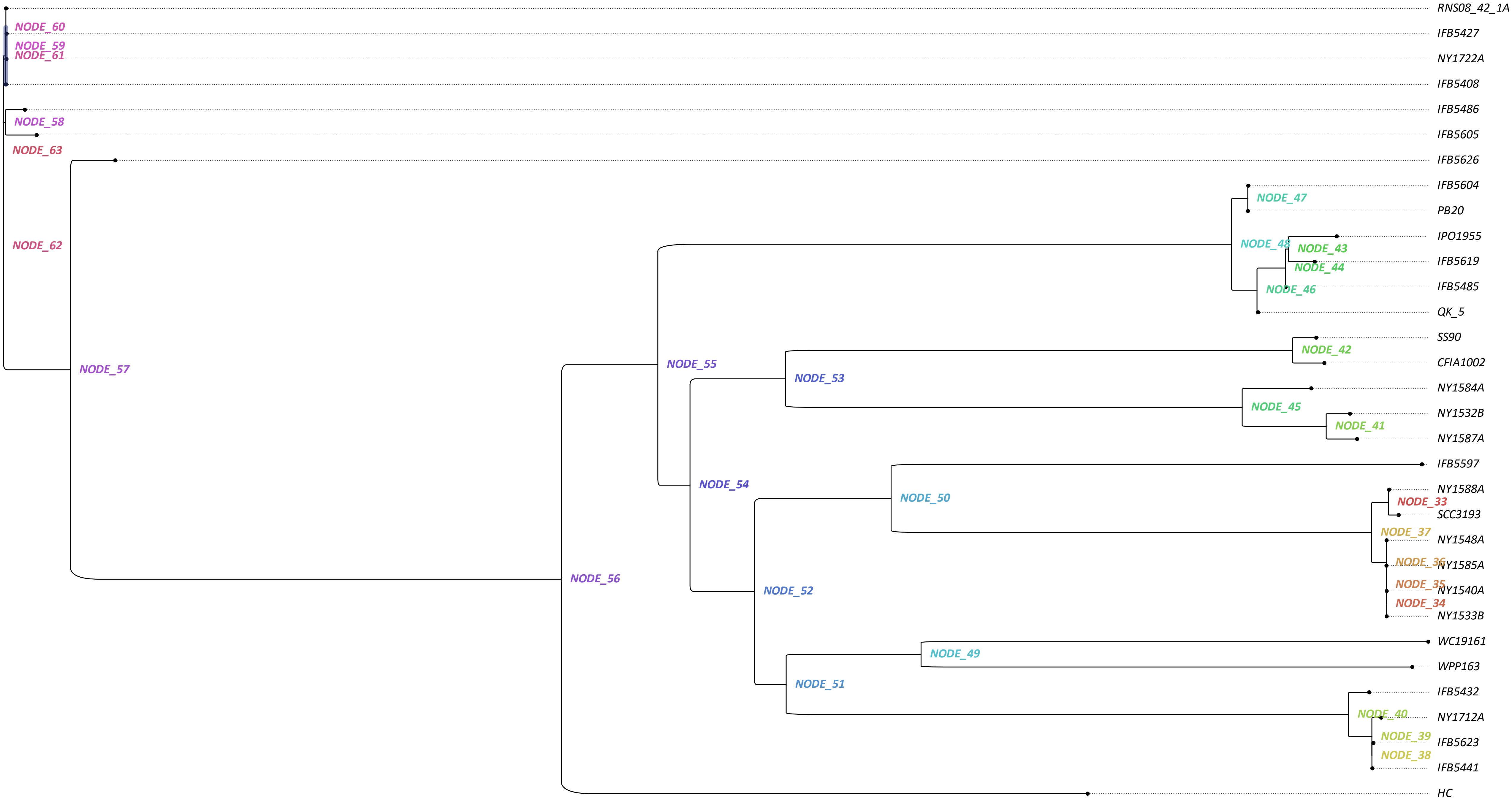


**Fig. S1**. Maximum likelihood tree based on the core genome alignment of 32 *P. parmentieri* strains. The positions and names of all nodes assigned by ClonalFrameML are indicated and highlighted with different colors. The tree was visualized using FigTree v1.4.4 and mid-point rooted.

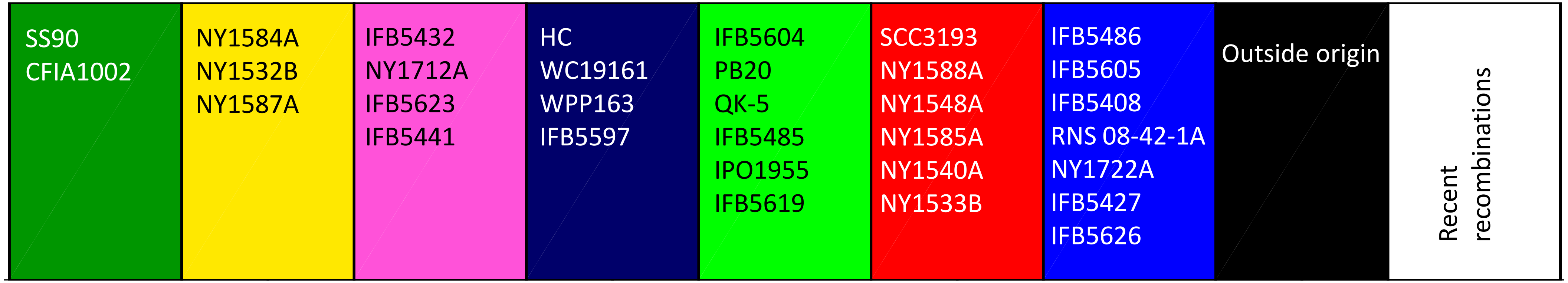


**Fig. S2**. Recombination plot. The image is depicting all colors used in the plotted recent and ancestral recombinations. Each color is related to each lineage and is labeled with their corresponding strains description. Black color indicates the outside origin, not corresponding to any of the lineages and white color indicates recent recombinations omitted in the inference of ancestral recombination analysis.
